# Factors Associated With Cause-specific Mortality in Older Patients With Advanced NSCLC Treated With PD-1 Inhibitors: A U.S. Population-based Cohort Study

**DOI:** 10.1177/10732748251380932

**Published:** 2025-10-03

**Authors:** Yeijin Kim, Yan Liu, Hae Sun Suh, Chanhyun Park

**Affiliations:** 1 College of Pharmacy, 26723 Kyung Hee University, Seoul, Republic of Korea; 2 Health Outcomes Division, College of Pharmacy, 12330 The University of Texas at Austin, Austin, TX, USA; 3 Institute of Regulatory Innovation Through Science (IRIS), 26723 Kyung Hee University, Seoul, Republic of Korea; 4 Department of Internal Medicine, Dell Medical School, 12330 The University of Texas at Austin, Austin, TX, USA; 5 Department of Regulatory Science, Graduate School, 26723 Kyung Hee University, Seoul, Republic of Korea

**Keywords:** NSCLC, immune checkpoint inhibitors, PD-1 inhibitors, cause of death, CVD mortality, SEER, cardio-oncology, geriatric oncology

## Abstract

**Introduction:**

Despite improved outcomes with PD-1 inhibitors (PD-1i) in advanced non–small cell lung cancer (NSCLC), the determinants of cancer-, cardiovascular disease (CVD)-, and other-cause mortality remain poorly characterized in older adults. This study applies competing-risks methods to estimate cause-specific mortality and to identify predictors for each cause in this population.

**Methods:**

A retrospective cohort study was conducted using the 2014-2019 SEER-Medicare linked database, including patients aged 65 years or older with advanced NSCLC who received PD-1i (nivolumab or pembrolizumab). Mortality outcomes included deaths from CVD, NSCLC, other cancers, and other diseases. Predictors included treatment-related factors (e.g., PD-1i type), demographic factors (e.g., age, sex), socioeconomic status (e.g., Medicaid dual eligibility), cancer-related factors (e.g., NSCLC stage), and comorbidities (e.g., congestive heart failure [CHF]). Competing risk analyses were performed using the Fine-Gray model, with cause-specific Cox models for sensitivity analyses.

**Results:**

Among 5076 patients, 68.36% received nivolumab and 31.64% received pembrolizumab. Of 3746 deaths, most were from NSCLC (85.34%), while 2.80% were from CVD. No significant difference in CVD mortality risk was observed between the two PD-1i (sub-distribution HR [sHR] = 1.08; 95% CI: 0.63-1.84), but NSCLC mortality was associated with a lower risk in the pembrolizumab group (sHR = 0.67; 95% CI: 0.60-0.74) compared to the nivolumab group. A history of CHF (sHR = 2.10; 95% CI: 1.37-3.21) and Medicaid dual eligibility (sHR = 2.70 vs private insurance; 95% CI: 1.28-5.56) were associated with increased CVD mortality. NSCLC mortality was higher in Stage IV/distant than in Stage IIIB/regional (sHR = 1.24; 95% CI: 1.13-1.35) and males (sHR = 1.11; 95% CI: 1.03-1.20).

**Conclusions:**

These results highlight the potential value of integrated cardio-oncology models and strategies addressing socioeconomic inequities among older adults receiving PD-1i. However, conclusions should be tempered by the SEER–Medicare data structure, including lack of biomarker information and restricted applicability to younger or non-Medicare populations.

## Plain language summary

Lung cancer remains the leading cause of cancer-related deaths in the U.S. Thanks to new treatments like immunotherapy, especially for a type called non-small cell lung cancer (NSCLC), more patients are living longer. Two commonly used immunotherapy drugs—nivolumab and pembrolizumab—have shown promising results. However, there are concerns about side effects, including risks to heart health. This is especially important for older adults, who often already have heart conditions. This study looked at nearly 5000 people aged 65 or older with advanced NSCLC who were treated with either nivolumab or pembrolizumab. Researchers used the national cancer registry and Medicare records to understand which causes of death were most common, such as NSCLC, heart disease, or other diseases, and which patient factors were linked to these outcomes. Most patients died from NSCLC, while a small number died from heart-related problems. The risk of heart-related death was similar between the two drugs. The study also found that patients who had a history of congestive heart failure or were enrolled in both Medicare and Medicaid (a national health insurance program for people with lower income) were more likely to die from heart disease. Lung cancer deaths were more common among men and those with more advanced stages of cancer. These findings suggest that when treating older adults with lung cancer, doctors should consider more than just the cancer. A patient’s heart health and financial situation may also affect treatment outcomes. This information can help guide more personalized treatment decisions and better support the needs of older adults receiving immunotherapy.

## Introduction

Lung cancer is the second most commonly diagnosed cancer and remains the leading cause of cancer-related deaths in the United States, accounting for approximately 20% of all cancer deaths.^1^ More than 70% of lung cancer cases are diagnosed at advanced stages, classified as regional or distant, with a significant proportion occurring in individuals aged 65 years or older. The majority (approximately 84%) of lung cancer cases are non-small cell lung cancer (NSCLC), and the median age at diagnosis is 71 years.^2,3^

Over the past decade, immune checkpoint inhibitors (ICIs), particularly those targeting programmed death-1 (PD-1), have significantly improved clinical outcomes in advanced NSCLC.^4^ Despite these advancements, ICIs are associated with immune-related adverse events (irAEs),^5^ and recent studies have reported that immune-mediated cardiovascular toxicities, such as myocarditis, though rare, are increasingly recognized and can be life-threatening.^6-9^ Given the large number of patients eligible for ICI therapy, recent studies emphasized the need for improved recognition, definitive clinical evidence of ICI-associated cardiovascular toxicities.^7-9^

Age is the most significant risk factor for cardiovascular disease (CVD),^10^ and older patients are more susceptible to cancer therapy-induced cardiotoxicity than younger counterparts.^11^ In NSCLC, non-cancer-specific mortality is a major competing risk for NSCLC-specific mortality, and its contribution increases with age.^12,13^ Notably, deaths from non-NSCLC causes rise progressively with age, with CVD being the most prominent contributor.^13^ However, prior studies focused on early-stage disease or did not account for ICI treatment. Furthermore, despite the increasing number of older patients with NSCLC, they remain underrepresented in clinical trials, including those evaluating ICIs,^14,15^ resulting in limited population-level data on mortality patterns and their factors in this population.

With the advent of PD-1 inhibitors and other novel therapies, survival in advanced NSCLC has improved; nonetheless, 5-year relative survival remains low.^16^ As a result, the influence of comorbid conditions, both cancer-related and non–cancer-related, on outcomes has become increasingly salient. To individualize care and reduce preventable deaths, it is essential to delineate competing causes of death and their predictors. However, studies specifically characterizing cause-specific mortality among older adults with advanced NSCLC treated with PD-1 inhibitors remain limited. To address this critical knowledge gap, our study aims to examine the competing risks of cause-specific mortality in older patients with advanced NSCLC receiving PD-1 inhibitors. Specifically, we aim to identify factors influencing NSCLC-related and CVD-related mortality in this population.

## Methods

### Data Sources

The Surveillance, Epidemiology, and End Results (SEER)-Medicare linked database combines clinical, demographic, and cause-of-death information from cancer registries with claims data from the Medicare program.^17,18^ The SEER program, sponsored by the National Cancer Institute, collects cancer epidemiology data covering approximately 48% of the U.S. population.^19^ Medicare is a federal health insurance program serving older adults, individuals with disabilities, and those with end-stage renal disease, and it is the primary health insurance provider for 97% of the U.S. population aged 65 and older.^17^ The SEER-Medicare data do not include direct identifiers.^20^ The Institutional Review Board of the University of Texas at Austin determined that this study was not classified as human subjects research and was secondary use of a de-identified data set.

### Study Design

**Figure 1.**
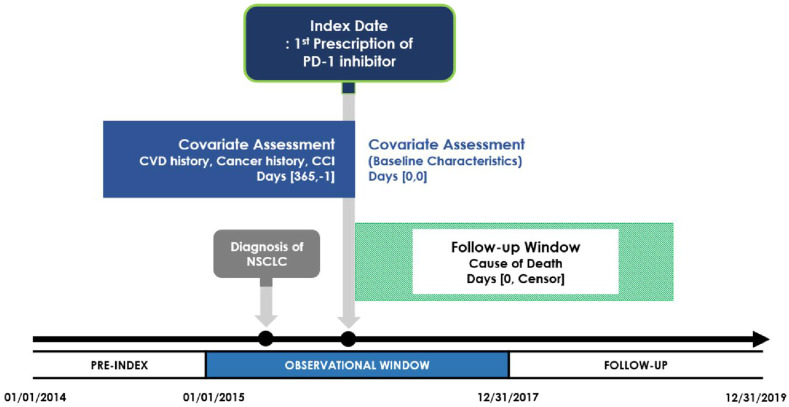
Study Design Abbreviations: PD-1 = Programmed Cell Death Protein 1, CVD = Cardiovascular Disease, CCI = Charlson Comorbidity Index, NSCLC = Non-small Cell Lung Cancer

This retrospective cohort study was conducted using the SEER-Medicare linked database from 2014 to 2019. The index date was defined as the initial receipt of pembrolizumab or nivolumab (Figure 1). The receipt of pembrolizumab or nivolumab was identified using the Healthcare Common Procedure Coding System (HCPCS) and National Drug Codes (NDC) (Supplemental Table 1). We defined the pre-index period as the 12 months before the index date to capture the patient’s history of comorbidities. The post-index period was defined as the follow-up period from the index date to the date of death or the end of the study (December 31, 2019), whichever occurred first. The reporting of this study conforms to STROBE guidelines.^21^

### Study Population

We included patients diagnosed with advanced NSCLC at age 65 years or older who received pembrolizumab or nivolumab between January 1, 2015, and December 31, 2017. NSCLC was defined using the International Classification of Diseases, tenth revision, Clinical Modification (ICD-10-CM: C34.x), and histologic type (ICD-O-3). We defined advanced NSCLC as stages IIIB to IV by the American Joint Committee on Cancer (AJCC) or regional and distant stages by the SEER summary stage.

Patients were excluded if they met any of the following criteria: (1) Eligibility for Medicare due to end-stage renal disease or disability during the study period, (2) Lack of continuous eligibility for both Medicare Parts A and B, (3) Enrollment in a Medicare health maintenance organization (HMO), (4) Death within 4 weeks of the index date, or (5) A discrepancy of more than two months between the date of death reported in SEER and Medicare.

### Outcomes

The primary outcomes of interest were overall survival and cause-specific mortality, classified into 4 groups: CVD, NSCLC, other cancers, and other diseases, based on the ICD-10-CM codes. CVD was defined as diseases of the heart, hypertension without heart disease, cerebrovascular diseases, atherosclerosis, aortic aneurysm, and other diseases of the arteries, arterioles, and capillaries, as identified using the ICD-10-CM codes.^22^

### Predictors

We included a range of variables, including demographics, tumor characteristics, treatment-related factors, and comorbid conditions, to assess their relationship with each cause of death. Information on patient age, gender, race, and socioeconomic factors such as income and geographic region was included. We also included the primary health insurance provider, marital status, stage of NSCLC at diagnosis, and detailed tumor characteristics, such as the primary site, laterality, and histology of NSCLC. Income was assessed at the area level using the median household income of the census tract in this study. Additionally, we included the Charlson Comorbidity Index (CCI) to evaluate the influence of pre-existing comorbidities,^23^ during the 1 year before the index date. Obesity, smoking, and alcohol use disorder history were also identified. We defined systemic corticosteroid use as an oral corticosteroid prescription exceeding 10 mg of prednisone-equivalent daily dosage after the index date.^24,25^

### Statistical Analysis

For the descriptive analysis of baseline characteristics, we used t-tests for continuous variables and Fisher’s exact test or chi-square test for categorical variables to compare characteristics between PD-1 inhibitor user groups. To estimate hazard ratios (HRs) for comparisons between two periods and the impact of each factor on death, we used the multivariate Cox proportional hazards regression model. Given that different cause-specific deaths were treated as competing risk events, the Fine-Gray model was used as the primary analysis to assess the relationship between predictors and mortality. Additionally, the cause-specific Cox model was employed as a sensitivity analysis to evaluate the robustness of the results. Statistical analyses were performed using SAS version 9.4 software (SAS Institute, Cary, NC, USA).

## Results

**Figure 2.**
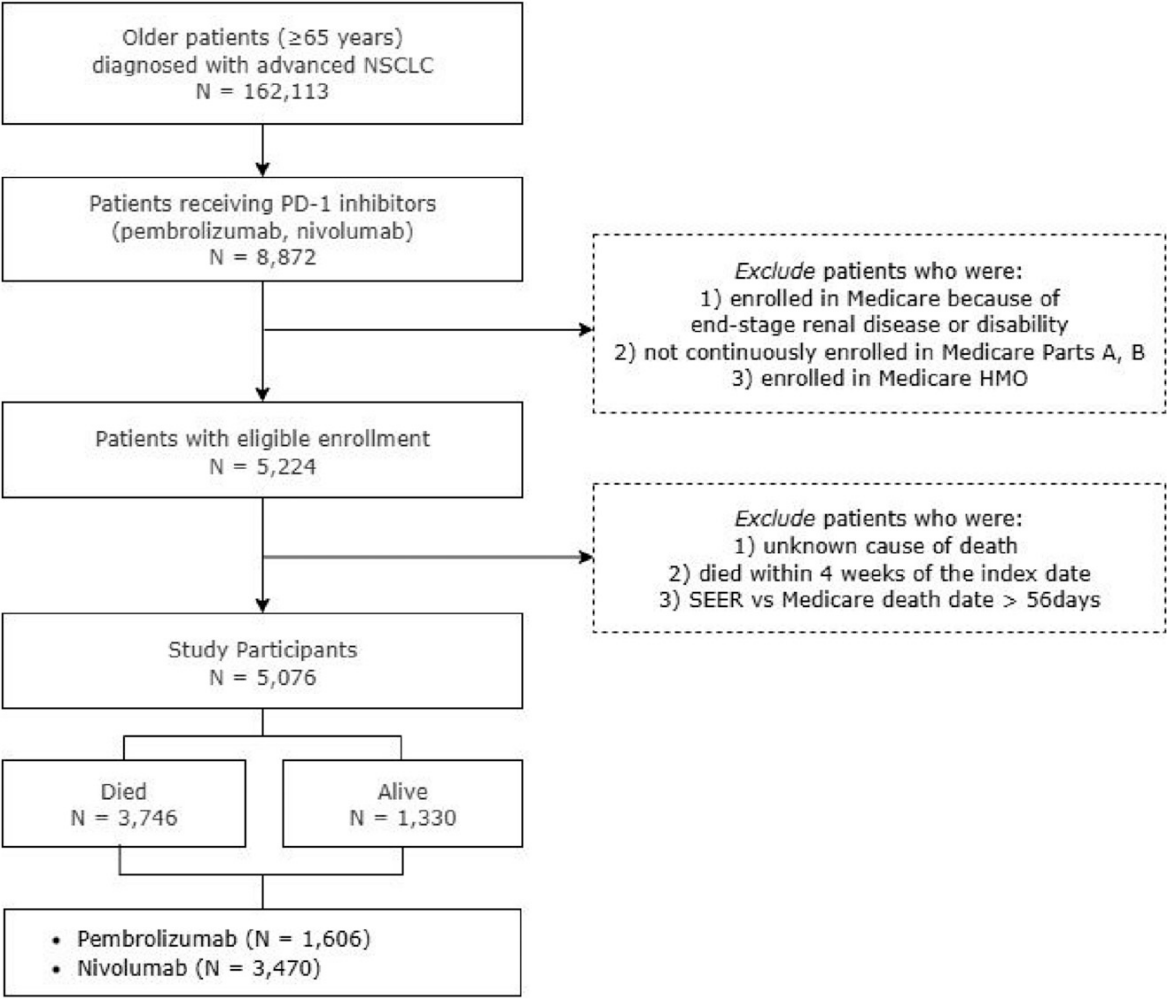
Patient Selection Flow Abbreviations: NSCLC = Non-small Cell Lung Cancer, PD-1 = Programmed Cell Death Protein-1, HMO = Health Maintenance Organization, SEER = Surveillance, Epidemiology, and End Results, CVD = Cardiovascular Disease

We identified 5076 older patients with advanced NSCLC who met the study’s inclusion criteria. Among them, 68.36% (n = 3470) were treated with nivolumab, and 31.64% (n = 1606) were treated with pembrolizumab (Figure 2). More than 70% of the participants (n = 3746) died during the study period. Among all the patients, the majority died from NSCLC (85.34%), while 1.17% of deaths were attributed to ischemic heart diseases (0.93% of total deaths in the pembrolizumab group and 0.84% in the nivolumab group) and 1.63% of deaths were attributed to other CVDs (1.25% of total deaths in the pembrolizumab group and 1.18% in the nivolumab group).

Table 1 shows the distribution of patient characteristics by PD-1 inhibitors. In both PD-1 inhibitor groups, the sex distribution was balanced (48.75% male in pembrolizumab group and 51.59% male in nivolumab group), and the mean age was approximately 76 years in both groups (76.40 ± 6.19 years in pembrolizumab group and 75.99 ± 5.85 years in nivolumab group; *P* = 0.02). While both groups were predominantly non-Hispanic White, this was slightly more common in the pembrolizumab group (84.43%) than in the nivolumab group (80.63%). A majority of patients had stage IV or distant cancer (78.46% in the pembrolizumab group and 69.71% in the nivolumab group) and adenocarcinoma (75.78% in the pembrolizumab group and 63.17% in the nivolumab group). We examined the predictors of overall mortality among older adults with NSCLC, estimated using a multivariate Cox proportional hazards model (Supplemental Table 2).

**Table 1.** Characteristics of Older Adults With Advanced Non-small Cell Lung Cancer (NSCLC) by Type of Programmed Death-1 (PD-1) Inhibitors (N = 5076)

Variables, n (%)	Pembrolizumab (N = 1606)	Nivolumab (N = 3470)	Total	*P*-value
Demographic factors				
Sex				
Male	783 (48.75)	1790 (51.59)	2573	0.0614^f^
Female	823 (51.25)	1680 (48.41)	2503	
Age group				
65–69	229 (14.26)	518 (14.93)	747	0.1810
70–74	440 (27.40)	1022 (29.45)	1462	
75–79	460 (28.64)	994 (28.65)	1454	
80+	477 (29.70)	936 (26.97)	1413	
Race				
Non-Hispanic White	1356 (84.43)	2798 (80.63)	4154	0.0088^f^
Non-Hispanic Black	88 (5.48)	261 (7.52)	349	
Hispanic	69 (4.30)	172 (4.96)	241	
Others^a^	93 (5.79)	239 (6.89)	332	
Geography				
Northeast	437 (27.21)	949 (27.35)	1386	0.0234
South	131 (8.16)	365 (10.52)	496	
Midwest	338 (21.05)	758 (21.84)	1096	
West	700 (43.59)	1398 (40.29)	2098	
Region				
Metropolitan	1389 (86.49)	3000 (86.46)	4389	0.9960
Urban	193 (12.02)	417 (12.02)	610	
Rural	24 (1.49)	53 (1.53)	77	
Marriage status				
Married	822 (51.18)	1978 (57.00)	2800	<0.0001
Non-married^b^	568 (35.37)	1185 (34.15)	1753	
Unknown	216 (13.45)	307 (8.85)	523	
Socioeconomic status				
Income level^c^				
Q1–Q2	750 (46.70)	1777 (51.21)	2527	0.0041
Q3–Q4	855 (53.24)	1693 (48.79)	2548	
Primary payer				
Medicaid	88 (5.48)	239 (6.89)	327	0.1381
Private insurance	471 (29.33)	1030 (29.68)	1501	
Others^d^	1047 (65.19)	2201 (63.43)	3248	
Cancer-related factors				
Year of diagnosis				
2007–2015	305 (18.99)	2482 (71.53)	2787	<0.0001
2016	448 (27.90)	807 (23.26)	1255	
2017	853 (53.11)	181 (5.22)	1034	
Stage				
IIIB/Regional	346 (21.54)	1051 (30.29)	1397	<0.0001^f^
IV/Distant	1260 (78.46)	2419 (69.71)	3679	
Histology				
Squamous	278 (17.31)	1062 (30.61)	1340	<0.0001
Adenocarcinoma	1217 (75.78)	2192 (63.17)	3409	
Large/Others	111 (6.91)	216 (6.22)	327	
Primary site				
Upper lobe, bronchus, or lung	813 (50.62)	1795 (51.73)	2608	0.4126
Middle lobe, bronchus, or lung	66 (4.11)	170 (4.9)	236	
Lower lobe, bronchus, or lung	467 (29.08)	951 (27.41)	1418	
Others	260 (16.19)	554 (15.97)	814	
Laterality				
Right	622 (38.73)	1371 (39.51)	1993	0.8667
Left	64 (3.99)	135 (3.89)	199	
Others^e^	920 (57.29)	1964 (56.6)	2884	
Treatment-related factors				
Radiation	591 (36.8)	1547 (44.58)	2138	<0.0001^f^
Surgery	140 (8.72)	428 (12.33)	568	0.0001^f^
Systemic corticosteroid	753 (46.89)	1859 (53.57)	2612	<0.0001
Comorbidities				
Charlson Comorbidity Index (CCI)				
CCI = 0	23 (1.43)	52 (1.5)	75	0.9332
0 < CCI < 9	644 (40.1)	1374 (39.6)	2018	
9 ≤ CCI	939 (58.47)	2044 (58.9)	2983	
Comorbidities				
Smoking	583 (36.3)	1360 (39.19)	1943	0.0505^f^
Prior cancer	754 (46.95)	1700 (48.99)	2454	0.1840
Myocardial infarction	199 (12.39)	376 (10.84)	575	0.1056^f^
Congestive heart failure	289 (18.00)	739 (21.30)	1028	0.0068^f^
Peripheral vascular disease	549 (34.18)	1221 (35.19)	1770	0.5061^f^
Cerebrovascular disease	427 (26.59)	877 (25.27)	1304	0.3335^f^
Dementia	55 (3.42)	102 (2.94)	157	0.3833^f^
COPD	1043 (64.94)	2367 (68.21)	3410	0.0225^f^
Rheumatic disease	89 (5.54)	159 (4.58)	248	0.1420^f^
Peptic ulcer disease	48 (2.99)	125 (3.60)	173	0.2803^f^
Mild liver disease	297 (18.49)	793 (22.85)	1090	0.0004^f^
Diabetes without chronic complication	472 (29.39)	1088 (31.35)	1560	0.1598^f^
Diabetes with chronic complication	199 (12.39)	399 (11.50)	598	0.3738^f^
Hemiplegia or paraplegia	47 (2.93)	71 (2.05)	118	0.0572^f^
Renal disease	304 (18.93)	716 (20.63)	1020	0.1636^f^
Moderate or severe liver disease	11 (0.68)	22 (0.63)	33	0.8519^f^
Metastatic solid tumor	1354 (84.31)	2845 (81.99)	4199	0.0419^f^

Abbreviations: SD = standard deviation, CCI = Charlson comorbidity index, COPD = chronic obstructive pulmonary disease.

^a^Others include American Indian/Alaska native/Asian/Pacific islander.

^b^Non-married include divorced/separated/widowed/never married.

^c^Income was assessed at the area level using the median household income of the census tract. Q1–Q2 indicates an income level ≤ $62,622, while Q3–Q4 indicates an income level > $62,622.

^d^Others include TRICARE/Military/Veterans Affairs/Indian/Public Health Service/Insurance status unknown.

^e^Others include origin unspecified/unknown/paired site.

^f^Fisher exact test was used.

The results from the multivariable Fine-Gray model showed no significant difference in mortality due to CVD (sub-distribution hazard ratio [sHR] = 1.08; 95% CI: 0.63-1.84; *P* = 0.79), other cancers (sHR = 0.92; 95% CI: 0.66-1.28; *P* = 0.63), or other diseases (sHR = 0.71; 95% CI: 0.48-1.05; *P* = 0.09) between the two PD-1 inhibitors (Supplemental Figure 1). However, a difference in NSCLC-related mortality was observed, with the pembrolizumab group showing a lower risk (sHR = 0.67; 95% CI: 0.60-0.74; *P* < 0.01) compared to the nivolumab group.

Medicaid beneficiaries had a significantly higher risk of CVD mortality compared to those with private insurance after controlling for other variables (sHR = 2.70 compared to private insurance; 95% CI: 1.28-5.56; *P* = 0.01, Table 2). NSCLC mortality was higher among patients with stage IV/distant cancer compared to those with stage IIIB/regional cancer (sHR = 1.24; 95% CI: 1.13-1.35; *P* < 0.01) and lower among females (sHR = 0.90; 95% CI: 0.83-0.97; *P* < 0.01). Histology type influenced NSCLC mortality, with adenocarcinoma (sHR = 0.90; 95% CI: 0.83-0.98; *P* = 0.01) showing lower mortality risk compared to squamous cell carcinoma.

**Table 2.** Competing Risks of Mortality in Older Patients With Advanced Non-small Cell Lung Cancer (NSCLC) Treated With Programmed Death-1 (PD-1) Inhibitors: Results From Fine-Gray Model

	Sub-distribution hazard ratios by cause of death			
	CVD sHR (95% CI)	NSCLC sHR (95% CI)	Other cancer sHR (95%CI)	Other diseases sHR (95%CI)
Demographic factors				
Sex				
Male	1.00	1.00	1.00	1.00
Female	1.04 (0.68-1.61)	0.90 (0.83-0.97)***	1.01 (0.77-1.32)	1.03 (0.75-1.42)
Age group				
65-69	1.00	1.00	1.00	1.00
70-74	1.93 (0.85-4.38)	1.05 (0.93-1.18)	0.77 (0.53-1.12)	1.14 (0.69-1.89)
75-79	2.20 (0.96-5.02)	1.11 (0.98-1.25)	0.90 (0.62-1.30)	1.17 (0.71-1.92)
80+	2.25 (0.98-5.13)	1.17 (1.04-1.32)*	0.94 (0.64-1.38)	1.11 (0.66-1.84)
Race				
Non-hispanic white	1.00	1.00	1.00	1.00
Non-hispanic black	0.66 (0.26-1.66)	1.00 (0.87-1.15)	1.26 (0.82-1.95)	0.86 (0.45-1.62)
Hispanic	1.05 (0.41-2.70)	0.91 (0.77-1.08)	1.04 (0.58-1.88)	1.30 (0.69-2.48)
Others^a^	0.99 (0.44-2.26)	0.89 (0.77-1.03)	1.09 (0.66-1.79)	1.50 (0.85-2.63)
Geography				
Northeast	1.00	1.00	1.00	1.00
South	1.98 (1.01-3.87)*	1.11 (0.96-1.27)	1.03 (0.60-1.75)	0.50 (0.27-0.94)*
Midwest	1.39 (0.74-2.63)	1.04 (0.92-1.16)	1.64 (1.07-2.51)*	0.62 (0.39-0.97)*
West	0.97 (0.55-1.71)	1.06 (0.96-1.17)	1.50 (1.02-2.19)*	0.61 (0.41-0.90)*
Marriage status				
Married	1.00	1.00	1.00	1.00
Non-married^b^	1.01 (0.66-1.56)	1.00 (0.93-1.08)	0.99 (0.75-1.31)	0.80 (0.57-1.13)
Socioeconomic status				
Income level^c^				
Q1-Q2 (≤$62,622)	1.00	1.00	1.00	1.00
Q3-Q4 (>$62,622)	1.31 (0.84-2.05)	1.02 (0.95-1.11)	0.87 (0.66-1.15)	0.76 (0.55-1.05)
Primary payer				
Medicaid	1.00	1.00	1.00	1.00
Private insurance	0.37 (0.18-0.78)**	1.11 (0.95-1.31)	0.99 (0.60-1.62)	0.74 (0.42-1.32)
Others^d^	0.51 (0.27-0.96)*	1.12 (0.96-1.30)	0.81 (0.51-1.29)	0.69 (0.40-1.19)
Cancer-related factors				
Year of diagnosis				
2007-2015	1.00	1.00	1.00	1.00
2016	1.41 (0.88-2.27)	1.04 (0.95-1.14)	0.88 (0.64-1.20)	0.84 (0.57-1.25)
2017	1.68 (0.91-3.10)	0.95 (0.83-1.08)	0.71 (0.46-1.08)	1.02 (0.62-1.66)
Stage				
Stage IIIB/Regional	1.00	1.00	1.00	1.00
Stage IV/Distant	0.67 (0.43-1.06)	1.24 (1.13-1.35)***	1.06 (0.78-1.45)	1.23 (0.84-1.80)
Histology				
Squamous	1.00	1.00	1.00	1.00
Adenocarcinoma	1.05 (0.66-1.68)	0.90 (0.83-0.98)*	0.88 (0.66-1.17)	0.81 (0.58-1.14)
Large/Others	0.98 (0.40-2.38)	0.88 (0.75-1.03)	1.01 (0.60-1.70)	0.89 (0.47-1.69)
Treatment-related factors				
PD-1 inhibitor				
Pembrolizumab	1.00	1.00	1.00	1.00
Nivolumab	1.08 (0.63-1.84)	1.49 (1.35-1.66)***	0.92 (0.66-1.28)	0.71 (0.48-1.05)
Radiation				
No	1.00	1.00	1.00	1.00
Yes	1.00 (0.66-1.53)	1.00 (0.93-1.08)	1.53 (1.17-2.01)**	1.43 (1.04-1.98)*
Surgery				
No	1.00	1.00	1.00	1.00
Yes	1.16 (0.62-2.18)	0.93 (0.82-1.05)	1.70 (1.15-2.51)**	1.09 (0.66-1.81)
Systemic corticosteroid use				
No	1.00	1.00	1.00	1.00
Yes	0.90 (0.60-1.33)	0.95 (0.89-1.02)	0.87 (0.68-1.11)	1.17 (0.86-1.58)
Comorbidities				
Smoking				
No	1.00	1.00	1.00	1.00
Yes	1.10 (0.72-1.70)	0.94 (0.88-1.02)	0.99 (0.77-1.29)	1.07 (0.78-1.48)
CHF				
No	1.00	1.00	1.00	1.00
Yes	2.10 (1.37-3.21)***	1.07 (0.97-1.17)	0.84 (0.60-1.16)	1.97 (1.40-2.78)***
MI				
No	1.00	1.00	1.00	1.00
Yes	1.55 (0.94-2.56)	1.12 (1.00-1.26)	1.27 (0.87-1.85)	0.76 (0.47-1.23)
PVD				
No	1.00	1.00	1.00	1.00
Yes	0.78 (0.52-1.16)	1.00 (0.93-1.08)	0.92 (0.70-1.22)	1.14 (0.83-1.57)
CBVD				
No	1.00	1.00	1.00	1.00
Yes	1.46 (0.96-2.22)	1.04 (0.96-1.12)	0.72 (0.53-0.99)*	1.02 (0.73-1.42)
COPD				
No	1.00	1.00	1.00	1.00
Yes	0.87 (0.55-1.38)	1.07 (0.99-1.16)	0.99 (0.75-1.31)	1.36 (0.95-1.97)
Mild liver disease				
No	1.00	1.00	1.00	1.00
Yes	0.88 (0.53-1.45)	1.06 (0.97-1.16)	1.06 (0.79-1.41)	0.85 (0.58-1.26)
Hemiplegia/paraplegia				
No	1.00	1.00	1.00	1.00
Yes	0.35 (0.05-2.51)	1.27 (1.00-1.61)	1.70 (0.85-3.39)	0.59 (0.19-1.85)

Abbreviations: HR = hazard ratio; CVD = cardiovascular disease; NSCLC = non-small cell lung cancer.

CI = confidence interval; PD-1 inhibitor = programmed death-1 inhibitor; CHF = congestive heart failure.

MI = myocardial infarction; PVD = peripheral vascular disease; CBVD = cerebrovascular disease.

COPD = chronic obstructive pulmonary disease.

*Notes.* *: *P* < .05; **: *P* < .01; ***: *P* < .001.

^a^Others include American Indian/Alaska native/Asian/Pacific islander.

^b^Non-married include divorced/separated/widowed/never married.

^c^Income was assessed at the area level using the median household income of the census tract.

^d^Others include TRICARE/Military/Veterans Affairs/Indian/Public Health Service/Insurance status unknown.

A history of CHF was associated with increased mortality from all causes except NSCLC, including a two-fold higher risk of CVD mortality (sHR = 2.10; 95% CI: 1.37-3.21; *P* < 0.01) and mortality due to other diseases (sHR = 1.97; 95% CI: 1.40-2.78; *P* < 0.01). Older age was a significant predictor of NSCLC mortality. Patients over 80 years old had a 17% higher risk of NSCLC mortality (sHR = 1.17 compared to 65-69 years group; 95% CI: 1.04-1.32; *P* = 0.01).

Other treatment-related factors were associated with mortality. A history of lung cancer surgery was associated with higher mortality from other cancers (sHR = 1.70; 95% CI: 1.15-2.51; *P* = 0.01). A history of radiation was associated with higher mortality from other cancers (sHR = 1.53; 95% CI: 1.17-2.01; *P* < 0.01) or other diseases (sHR = 1.43; 95% CI: 1.04-1.98; *P* = 0.03).

The sensitivity analysis using the cause-specific Cox model confirmed the primary findings, with no significant differences in mortality due to CVD, other cancers, or other diseases between PD-1 inhibitors (Table 3). However, pembrolizumab remained associated with lower NSCLC mortality (HR = 0.66; 95% CI: 0.60-0.73; *P* < 0.01) compared to nivolumab. Older age was a significant predictor of both CVD and NSCLC mortality. The cause-specific Cox model showed that patients over 80 years old had more than twofold higher of CVD mortality (HR = 2.50; 95% CI: 1.08-5.77; *P* = 0.03) and a 21% higher risk of NSCLC mortality (HR = 1.21; 95% CI: 1.08-1.36; *P* < 0.01) compared to 65-69 years group.

**Table 3.** Competing Risks of Mortality in Older Patients With Advanced Non-small Cell Lung Cancer (NSCLC) Treated With Programmed Death-1 (PD-1) Inhibitors: Results From Cause-specific Cox Model

	Cause-specific hazard ratios by cause of death			
	CVD HR (95% CI)	NSCLC HR (95% CI)	Other cancer HR (95%CI)	Other diseases HR (95%CI)
Demographic factors				
Sex				
Male	1.00	1.00	1.00	1.00
Female	0.99 (0.66-1.51)	0.90 (0.84-0.97)**	0.95 (0.73-1.22)	0.99 (0.72-1.37)
Age group				
65-69	1.00	1.00	1.00	1.00
70-74	2.11 (0.92-4.85)	1.07 (0.95-1.20)	0.80 (0.55-1.16)	1.16 (0.70-1.92)
75-79	2.46 (1.08-5.60)*	1.16 (1.03-1.30)*	1.00 (0.69-1.45)	1.26 (0.75-2.09)
80+	2.50 (1.08-5.77)*	1.21 (1.08-1.36)**	1.03 (0.70-1.51)	1.17 (0.69-1.98)
Race				
Non-hispanic white	1.00	1.00	1.00	1.00
Non-hispanic black	0.64 (0.25-1.60)	1.01 (0.88-1.16)	1.25 (0.80-1.93)	0.90 (0.48-1.69)
Hispanic	0.99 (0.39-2.53)	0.93 (0.78-1.10)	1.03 (0.58-1.84)	1.26 (0.66-2.39)
Others^a^	0.94 (0.41-2.14)	0.91 (0.79-1.06)	1.03 (0.63-1.67)	1.42 (0.80-2.53)
Geography				
Northeast	1.00	1.00	1.00	1.00
South	1.91 (0.96-3.81)	1.08 (0.95-1.24)	1.06 (0.63-1.81)	0.49 (0.27-0.91)*
Midwest	1.39 (0.73-2.64)	1.05 (0.93-1.17)	1.68 (1.12-2.53)*	0.61 (0.38-0.96)*
West	0.95 (0.54-1.65)	1.05 (0.96-1.16)	1.52 (1.05-2.20)*	0.60 (0.41-0.89)*
Marriage status				
Married	1.00	1.00	1.00	1.00
Non-married^b^	0.96 (0.62-1.47)**	0.98 (0.91-1.06)	0.97 (0.74-1.27)	0.76 (0.54-1.07)
Socioeconomic status				
Income level^c^				
Q1-Q2 (≤$62,622)	1.00	1.00	1.00	1.00
Q3-Q4 (>$62,622)	1.35 (0.88-2.08)	1.01 (0.94-1.09)	0.89 (0.68-1.15)	0.76 (0.55-1.05)
Primary payer				
Medicaid	1.00	1.00	1.00	1.00
Private insurance	0.35 (0.17-0.72)**	1.07 (0.91-1.25)	0.99 (0.61-1.62)	0.74 (0.41-1.33)
Others^d^	0.50 (0.27-0.95)*	1.08 (0.93-1.25)	0.83 (0.52-1.32)	0.71 (0.41-1.22)
Cancer-related factors				
Year of diagnosis				
2007-2015	1.00	1.00	1.00	1.00
2016	1.56 (0.96-2.54)	1.09 (1.00-1.19)	0.97 (0.71-1.32)	0.95 (0.64-1.42)
2017	1.80 (0.96-3.37)	0.99 (0.87-1.12)	0.79 (0.52-1.22)	1.14 (0.70-1.86)
Stage				
Stage IIIB/Regional	1.00	1.00	1.00	1.00
Stage IV/Distant	0.78 (0.49-1.24)	1.27 (1.16-1.39)***	1.19 (0.89-1.61)	1.40 (0.96-2.04)
Histology				
Squamous	1.00	1.00	1.00	1.00
Adenocarcinoma	0.97 (0.61-1.55)	0.89 (0.82-0.97)**	0.83 (0.63-1.10)	0.78 (0.55-1.10)
Large/Others	0.95 (0.39-2.33)	0.87 (0.74-1.02)	0.94 (0.56-1.56)	0.86 (0.46-1.62)
Treatment-related factors				
PD-1 inhibitor				
Pembrolizumab	1.00	1.00	1.00	1.00
Nivolumab	1.26 (0.75-2.11)	1.52 (1.38-1.68)***	1.11 (0.80-1.53)	0.82 (0.55-1.24)
Radiation				
No	1.00	1.00	1.00	1.00
Yes	1.06 (0.70-1.61)	1.05 (0.97-1.13)	1.62 (1.25-2.11)***	1.55 (1.13-2.14)**
Surgery				
No	1.00	1.00	1.00	1.00
Yes	1.19 (0.63-2.25)	0.97 (0.85-1.09)	1.75 (1.20-2.56)**	1.13 (0.66-1.92)
Systemic corticosteroid use				
No	1.00	1.00	1.00	1.00
Yes	0.73 (0.49-1.08)	0.90 (0.83-0.96)**	0.74 (0.58-0.94)*	0.98 (0.73-1.33)
Comorbidities				
Smoking				
No	1.00	1.00	1.00	1.00
Yes	1.06 (0.70-1.62)	0.94 (0.87-1.01)	0.97 (0.75-1.26)	1.05 (0.77-1.45)
CHF				
No	1.00	1.00	1.00	1.00
Yes	2.41 (1.56-3.72)***	1.14 (1.04-1.25)**	0.94 (0.67-1.31)	2.26 (1.61-3.16)***
MI				
No	1.00	1.00	1.00	1.00
Yes	1.65 (0.99-2.74)	1.17 (1.04-1.31)**	1.45 (0.99-2.14)	0.83 (0.51-1.35)
PVD				
No	1.00	1.00	1.00	1.00
Yes	0.76 (0.50-1.16)	0.99 (0.92-1.07)	0.92 (0.70-1.20)	1.14 (0.83-1.56)
CBVD				
No	1.00	1.00	1.00	1.00
Yes	1.51 (1.00-2.28)*	1.04 (0.96-1.13)	0.73 (0.53-0.99)*	1.06 (0.75-1.49)
COPD				
No	1.00	1.00	1.00	1.00
Yes	0.94 (0.60-1.49)	1.10 (1.02-1.19)	1.07 (0.82-1.42)	1.46 (1.01-2.10)*
Mild liver disease				
No	1.00	1.00	1.00	1.00
Yes	0.90 (0.55-1.47)	1.07 (0.98-1.17)	1.14 (0.86-1.53)	0.87 (0.59-1.28)
Hemiplegia/paraplegia				
No	1.00	1.00	1.00	1.00
Yes	0.44 (0.06-3.21)	1.29 (1.02-1.61)*	2.01 (1.01-3.99)	0.65 (0.20-2.08)

Abbreviations: HR = hazard ratio; CVD = cardiovascular disease; NSCLC = non-small cell lung cancer.

CI = confidence interval; PD-1 inhibitor = programmed death-1 inhibitor; CHF = congestive heart failure.

MI = myocardial infarction; PVD = peripheral vascular disease; CBVD = cerebrovascular disease.

COPD = chronic obstructive pulmonary disease.

*Notes.* *P < .05; **P < .01; ***P < .001.

^a^Others include American Indian/Alaska native/Asian/Pacific islander.

^b^Non-married include divorced/separated/widowed/never married.

^c^Income was assessed at the area level using the median household income of the census tract.

^d^Others include TRICARE/Military/Veterans Affairs/Indian/Public Health Service/Insurance status unknown.

## Discussion

This retrospective study examined the competing risks of 4 causes of death among older adults with advanced NSCLC receiving nivolumab or pembrolizumab and identified factors influencing mortality. As expected in a population with late-stage NSCLC, the leading cause of death was NSCLC, which aligns with the previous study showing the risk of death from lung cancer increases with stage.^13^

We found no significant differences between the two PD-1 inhibitors in mortality related to CVD, other cancers, or other diseases after controlling for covariates. By contrast, pembrolizumab was associated with significantly lower NSCLC-related mortality compared to nivolumab. Although the pembrolizumab group had a higher proportion of stage IV disease, the nivolumab group had a higher prevalence of other adverse prognostic factors, including race/ethnicity, lower income, squamous histology, and several comorbidities.^26,27^ These variables were included in the survival model, yet the nivolumab group still showed a higher hazard of NSCLC-related death, suggesting potential selection bias or residual confounding beyond measured covariates. Prior randomized trials reported no meaningful differences in efficacy or safety between nivolumab and pembrolizumab in advanced NSCLC,^28,29^ and real-world studies also found no significant difference in progression-free survival after adjusting for covariates including programmed death-ligand 1 (PD-L1) expression.^30,31^ During the study period, pembrolizumab use was enriched for PD-L1–positive tumors by FDA label requirements,^32^ whereas nivolumab could be prescribed regardless of PD-L1 status.^33^ Accordingly, unmeasured differences in PD-L1 expression between treatment groups, which was not available in SEER-Medicare, could bias comparative estimates of NSCLC-specific mortality. Histology was also significant factor of NSCLC-related mortality, with adenocarcinoma and large cell carcinoma demonstrating a lower mortality risk compared to squamous cell carcinoma. These findings are consistent with previous research, which reporting that adenocarcinoma patients had significantly better 5-year overall survival rates than those with squamous cell carcinoma.^34^

We found that certain patient demographics were associated with specific causes of death. As age is a well-established risk factor for mortality,^12,13^ our findings confirmed that older age was associated with higher NSCLC mortality. In the primary analysis using the Fine-Gray model, age showed a non-significant trend toward higher CVD mortality; however, the sensitivity analysis demonstrated a significantly higher hazard for CVD mortality among older patients. This association was particularly pronounced in patients aged 80 years or older compared to the younger group, emphasizing the need for tailored geriatric care in oncology. Sex-based differences in outcomes were also notable, with male patients exhibiting higher NSCLC mortality. This aligns with prior research indicating that female sex is a consistent predictor of better survival outcomes, independent of histology, stage, or treatment.^35-37^ These results highlight the importance of understanding sex-based differences in response to cancer therapies. In contrast to previous reports,^14,27^ neither Hispanic nor Black race/ethnicity was not significantly associated with mortality from any cause in our study. This may be attributable to the predominance of White patients (over 80%), and limited statistical power for subgroup comparisons.

Furthermore, clinical factors were associated with mortality. Pre-existing congestive heart failure (CHF) was associated with a significantly increased risk of not only CVD mortality but also mortality due to NSCLC and other diseases. CHF is one of the most common comorbid conditions in NSCLC patients and is associated with a significantly increased mortality risk.^38-40^ A German study also reported that the prevalence of CHF increases with age.^39^ The elevated mortality risk may be due to the lower likelihood of NSCLC patients with CHF receiving surgery or chemotherapy compared to those without.^40^ These findings underscore the importance of integrating routine cardiovascular screening and management strategies into the care of older NSCLC patients receiving ICIs to address overlapping risks.

Treatment-related adverse events, particularly systemic steroid use can affect survival. In our primary analysis, systemic corticosteroid use was not associated with mortality from any cause, whereas the sensitivity analysis suggested a protective effect on NSCLC- and other cancer-related mortality. Corticosteroids are often used at higher doses for cancer-related symptoms^41^; however, their role during ICI treatment remains debated. Some studies reported the systemic corticosteroid use was related to poor outcomes in patients with NSCLC,^42,43^ while others reported detrimental effects only when used for cancer-related palliative indications, with no association for non cancer-related use.^44^

Socioeconomic factors were also related to mortality. Medicare-Medicaid dual beneficiaries had an increased risk of CVD mortality after adjusting for covariates. This population is more likely to have low income and a high disease burden,^45,46^ and socioeconomic factors can influence access to care, treatment adherence, and outcomes.^26,27,45,47,48^ Survival disparities associated with Medicaid status are well-documented, although prior studies often did not specify cause of death or treatment type.^49-51^ Insurance-related disparities in CVD mortality among cancer survivors have also been reported. A recent study using SEER data found that lung cancer patients without insurance or on Medicaid had significantly higher CVD mortality than those with non-Medicaid insurance.^38^ Lower PD-L1 testing rate and among NSCLC patients on Medicaid compared to those with commercial insurance may further contribute to these disparities.^51^ Our findings confirm persistent insurance-related survival disparities in NSCLC patients, particularly in CVD mortality, highlighting the need for integrated cancer and CVD management in this vulnerable population.

This study has several limitations. First, the SEER-Medicare linked database does not include laboratory biomarkers, including PD-L1 expression levels, which may contribute to residual confounding.^52^ Furthermore, although SEER-Medicare data contain variables related to behavioral factors such as smoking, alcohol consumption, and obesity, these variables have low sensitivity and may be underreported.^53^ Additionally, a recent study suggests that the “Primary Payer at Diagnosis” variable in the SEER dataset may be unreliable.^54^ Since all patients in our study were expected to be insured through Medicare by definition, this variable may have limitations in reflecting differences in insurance coverage. Second, the study period may limit the generalizability of our findings to the current population. We identified patients who received PD-1 inhibitors between 2015 and 2017. Around 2017, ICI-induced myocarditis became more widely recognized and systematically managed. Since then, significant advancements have been made in understanding, diagnosing, and managing cardiotoxic AEs, including myocarditis. Consequently, our findings may not fully reflect the current clinical practice in cardio-oncology. Third, our study did not include data on treatment line or duration, which may limit the interpretation of efficacy and toxicity outcomes. Both PD-1 inhibitors received initial FDA approval in 2015 for metastatic NSCLC in the second- or later-line setting,^32,33^ and contemporaneous clinical guidelines likewise recommended them for second-line treatment.^55,56^ Given the study’s identification window (2015-2017), it can be assumed that most patients received PD-1 inhibitors in the second-line or later setting. Fourth, while newer ICIs, such as PD-L1 and cytotoxic T-lymphocyte–associated protein 4 (CTLA-4) inhibitors, have recently been approved, their limited use in this cohort precluded meaningful analysis. As a result, our study focused exclusively on two PD-1 inhibitors. Future research using more recent data could provide a broader understanding of ICIs and their impact on mortality and causes of death in patients with NSCLC. Lastly, the study population consists exclusively of Medicare beneficiaries aged 65 years or older, limiting the generalizability of the findings to younger or non-Medicare populations.

Despite these limitations, our study has several notable strengths. First, to our knowledge, this is the first study to investigate overall survival and four types of cause-specific mortality among patients receiving PD-1 inhibitors in a real-world, population level in the U.S. Second, by categorizing mortality into four specific causes, including CVD, we provide valuable insights into the importance of managing comorbidities and risk factors in older adults with advanced NSCLC. Lastly, our use of both Fine-Gray and cause-specific Cox models ensures robust handling of competing risk events, consistent with best practices in competing risk analysis.^57^

## Conclusions

While PD-1 inhibitors have demonstrated significant survival benefits in patients with advanced NSCLC, their immune-related cardiotoxic effects are an increasing concern. However, their impact on cause-specific mortality, including CVD mortality remains poorly understood. We found no significant differences in CVD mortality between the two PD-1 inhibitors, though pembrolizumab was associated with lower NSCLC mortality. A history of CHF and Medicaid dual eligibility were significant risk factors for CVD mortality, highlighting the need for effective management of cardiovascular comorbidities in patients with advanced NSCLC, particularly in vulnerable populations. Additionally, older age was identified as a strong predictor of significantly higher CVD and NSCLC mortality, emphasizing the critical importance of integrating geriatric cardio-oncology into patient care. However, conclusions should be tempered by the SEER–Medicare data structure, including lack of biomarker information and restricted applicability to younger or non-Medicare populations.

## Supplemental Material


Supplemental material - Factors Associated With Cause-specific Mortality in Older Patients With Advanced NSCLC Treated With PD-1 Inhibitors: A Retrospective Study
Supplemental material for Factors Associated With Cause-specific Mortality in Older Patients With Advanced NSCLC Treated With PD-1 Inhibitors: A Retrospective Study by Yeijin Kim, PharmD MS, Yan Liu, MD, PhD, Hae Sun Suh, MA, PhD, Chanhyun Park, MEd, PhD in Cancer Control

## Data Availability

The data supporting this study’s findings were obtained from SEER-Medicare and are subject to restrictions. These data were used under a license specific to this study and are not publicly available. Access to the data can be requested through SEER-Medicare at https://healthcaredelivery.cancer.gov/seermedicare/obtain/, with appropriate permissions.

## Appendix

### Abbreviations

NSCLC: Non-Small Cell Lung Cancer ICI: Immune Checkpoint InhibitorPD-1: Programmed Death-1FDA: Food and Drug Administrationir AEs: immune-related Adverse Events WHOL World Health Organization CVD: Cardiovascular Disease SEER: Surveillance, Epidemiology, and End Results HCPCS: Healthcare Common Procedure Coding System NDC: National Drug Codes ICD-10-CM: International Classification of Diseases, tenth revision, Clinical Modification AJCC: American Joint Committee on Cancer HMO: Health Maintenance Organization CCI: Charlson Comorbidity Index HR: Hazard Ratios HR: sub-distribution Hazard Ratio PD-L1: Programmed Death-Ligand 1: ICI: Immune Checkpoint Inhibitor PD-1: Programmed Death-1FDA: Food and Drug AdministrationirAEs: immune-related Adverse Events WHOL World Health Organization CVD: Cardiovascular Disease SEER: Surveillance, Epidemiology, and End Results HCPCS: Healthcare Common Procedure Coding System NDC: National Drug Codes ICD-10-CM: International Classification of Diseases, tenth revision, Clinical Modification AJCC: American Joint Committee on Cancer HMO: Health Maintenance Organization CCI: Charlson Comorbidity Index HR: Hazard Ratios HR: sub-distribution Hazard Ratio PD-L1: Programmed Death-Ligand 1CTLA-4: Cytotoxic T-Lymphocyte–Associated protein 4.
