# Supplementary material for: Factors Associated With Cause-specific Mortality in Older Patients With Advanced NSCLC Treated With PD-1 Inhibitors: A U.S. Population-based Cohort Study
Source: Cancer Control. 2025 Oct 3;32:10732748251380932. doi: 10.1177/10732748251380932 (PMC12495207; doi:10.1177/10732748251380932)
Supplement: Supplemental material - Factors Associated With Cause-specific Mortality in Older Patients With Advanced NSCLC Treated With PD-1 Inhibitors: A Retrospective Study [file sj-pdf-1-ccx-10.1177_10732748251380932.pdf]

Supplementary Table 1 Code definitions for programmed death-1 (PD-1) inhibitors

Programmed death-1 (PD-1) inhibitors	Codes
Nivolumab	HCPCS: C9453, J9299<br>NDC: 00002121711, 00003373413, 00003377211, 00003377412, 00003375614
Pembrolizumab	HCPCS: C9027, J9271<br>NDC: 00006302601, 00006302602, 00006302604, 00006302901, 00006302902

Abbreviations: HCPCS, healthcare common procedure coding system; NDC, national drug code.

Supplementary Table 2 All-cause mortality in older patients with advanced non-small cell lung cancer (NSCLC) treated with programmed death-1 (PD-1) inhibitors: Results from multivariable Cox proportional hazards model

	**All-Cause Mortality Adjusted HR (95% CI)**
Demographic factors	
Sex	
Male	1.00
Female	0.91 (0.85-0.98)**
Age Group	
65-69	1.00
70-74	1.06 (0.95-1.18)
75-79	1.17 (1.05-1.30)**
80+	1.21 (1.08-1.35)***
Race	
Non-Hispanic White	1.00
Non-Hispanic Black	1.01 (0.89-1.15)
Hispanic	0.95 (0.82-1.12)
Others[a]	0.94 (0.82-1.08)
Geography	
Northeast	1.00
South	1.06 (0.94-1.20)
Midwest	1.06 (0.96-1.18)
West	1.05 (0.96-1.14)
Marriage Status	
Married	1.00
Non-married[b]	0.97 (0.90-1.04)
Socioeconomic status	
Income Level[c]	
Q1-Q2 (≤ $62,622)	1.00
Q3-Q4 (> $62,622)	1.00 (0.93-1.07)
Primary Payer	
Medicaid	1.00
Private insurance	1.00 (0.87-1.16)
Others[d]	1.00 (0.88-1.14)
Cancer-related factors	
Year of Diagnosis	
2007-2015	1.00
2016	1.09 (1.00-1.18)*
2017	1.00 (0.89-1.12)
Stage	
Stage IIIB/Regional	1.00
Stage IV/Distant	1.25 (1.15-1.36)***
Histology	
Squamous	1.00
Adenocarcinoma	0.88 (0.82-0.95)**
Large/Others	0.88 (0.76-1.01)

Treatment-related factors	
PD-1 inhibitor	
Pembrolizumab	1.00
Nivolumab	1.43 (1.31-1.57)***
Radiation	
No	1.00
Yes	1.10 (1.02-1.18)**
Surgery	
No	1.00
Yes	1.03 (0.91-1.15)
Systemic corticosteroid use	
No	1.00
Yes	0.88 (0.83-0.94)***
Comorbidities	
Smoking	
No	1.00
Yes	0.95 (0.89-1.02)
CHF	
No	1.00
Yes	1.20 (1.10-1.30)***
MI	
No	1.00
Yes	1.18 (1.07-1.31)**
PVD	
No	1.00
Yes	0.99 (0.92-1.06)
CBVD	
No	1.00
Yes	1.03 (0.95-1.11)
COPD	
No	1.00
Yes	1.11 (1.03-1.19)**
Mild liver disease	
No	1.00
Yes	1.06 (0.98-1.15)
Hemiplegia/paraplegia	
No	1.00
Yes	1.27 (1.03-1.56)*

Abbreviations: sHR = sub-distribution hazard ratio, CVD = cardiovascular disease,
NSCLC = non-small cell lung cancer, CI = confidence interval, PD-1 inhibitor = programmed death-1 inhibitor, CHF = congestive heart failure, MI = myocardial infarction, PVD = peripheral vascular disease, CBVD = cerebrovascular disease, COPD = chronic obstructive pulmonary disease.
Notes: *: p<.05; **: p<.01; ***: p<.001.
[a] Others include American Indian/Alaska native/Asian/Pacific islander.
[b] Non-married include divorced/separated/widowed/never married.
[c] Income was assessed at the area level using the median household income of the census tract.
[d] Others include TRICARE/Military/Veterans Affairs/Indian/Public Health Service/Insurance status unknown.

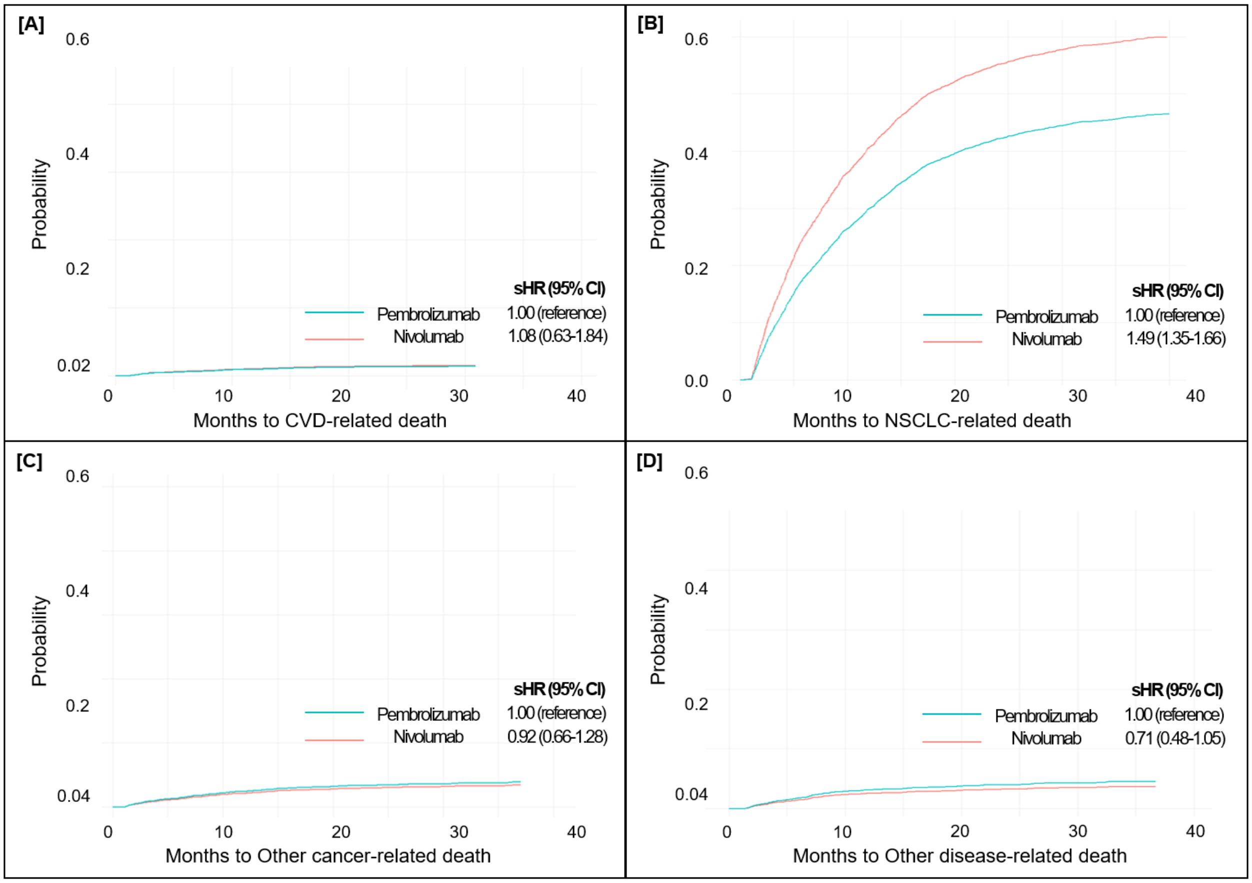

Supplementary Figure 1 Cumulative incidence function curves from multivariable adjusted Fine-Gray models: (A) CVD mortality; (B) NSCLC mortality; (C) Other cancer-related mortality; (4) Other diseases-related mortality

Abbreviations: sHR=sub-distribution hazard ratio, CI=confidence interval, CVD=cardiovascular disease, NSCLC=non-small cell lung cancer.
